# Draft Genome Sequence of Bacillus pacificus KVCMST-8A-12, Isolated from a Marine Sediment Sample from the Kanyakumari Coast, India

**DOI:** 10.1128/MRA.01011-21

**Published:** 2021-12-16

**Authors:** Asha Santhi, Venkatesh Subramanian, Krishnaveni Muthan

**Affiliations:** a Immuno-Pharmacology Laboratory, Center for Marine Science and Technology, Manonmaniam Sundaranar University, Marina Campus, Rajakkamangalam, Kanyakumari District, Tamil Nadu, India; b Genetic Engineering and Regenerative Biology Laboratory, Department of Biotechnology, Manonmaniam Sundaranar University, Tirunelveli, Tamil Nadu, India; Montana State University

## Abstract

A DNase-producing Bacillus pacificus strain was isolated, and the whole-genome sequence is reported in this paper. The draft genome sequence of Bacillus pacificus KVCMST-8A-12 constitutes 2.4 Gbp of raw reads, with a GC content of 35.24%. In total, 5,661 protein-coding genes, 64 tRNA genes, and 4 rRNA genes were predicted.

## ANNOUNCEMENT

A spore-forming, rod-shaped, aerobic bacterium designated Bacillus pacificus KVCMST-8A-12 was isolated from a sediment sample from the coastal region in Kanyakumari, India (1). This strain was reported to produce extracellular DNase, which can be commercially exploited (1). The whole genome of B. pacificus KVCMST-8A-12 was sequenced. Briefly, genomic DNA was extracted and purified from B. pacificus KVCMST-8A-12, which had been grown at 32°C in Zobell marine broth, using a QIAmp DNA extraction kit (Qiagen) and following the manufacturer’s instructions. DNA quantity and quality were assessed using NanoDrop measurements at 260 nm and 280 nm and resolution in a 0.8% agarose gel, respectively. The library was prepared following the workflow of the TruSeq DNA library preparation kit (Illumina) (2). The library was sequenced with a high-throughput sequencer (Illumina HiSeq 2500 sequencer) using paired-end sequencing strategies with 150-bp paired-end sequencing.

A total of 2.4 Gb of raw data was generated from 8,053,402 raw reads for B. pacificus KVCMST-8A-12; the reads were assembled using SPAdes v3.14.1 (3) and FastQC v0.11.8 (4) after filtering of low-quality reads (Q scores of ≤5) and adaptor sequences using Trimmomatic v0.38 (5). Reads were polished using Pilon v1.23 and Unicycler v0.4.9 (6). A genome size of 5,508,258 bp annotated with 383 scaffolds and 411 contigs (*N*_50_ value of 219,973 bp), with a GC content of 35.24% and 100× coverage, was obtained for our strain. The online RAST gene annotation pipeline (7) predicted 5,661 protein-coding genes. The annotation included 1,474 hypothetical proteins and 4,187 proteins with functional assignments. The proteins with functional assignments included 1,093 proteins with Enzyme Commission (EC) numbers (8), 920 proteins with Gene Ontology (GO) assignments (9), and 810 proteins mapped to KEGG pathways (10). PATRIC v3.6.12 annotation includes two types of protein families (11, 12). This genome has 5,547 proteins that belong to cross-genus protein families (PGFams). The average protein length determined by Prokka was 276 amino acids, and 28 rRNAs, 1 transfer-messenger RNA, and 77 tRNAs were obtained through Prokaryotic Genome Annotation Pipeline (PGAP) annotation. When annotation with Prokka v1.12 (13) and annotation with NCBI PGAP (14) were compared with RAST annotation, a few differences were noted. This may be due to different parameters and databases used by these three tools. Further annotation is in progress to identify the genes responsible for DNase production, which could find utility in various bioscience fields. Default parameters were used for all software unless otherwise noted.

### Data availability.

The whole-genome sequence of B. pacificus KVCMST-8A-12 has been deposited in GenBank under BioProject accession number PRJNA656513 with BioSample accession number SAMN16200492, and raw reads can be found under SRA accession number SRX9149524.
